# Molecular cloning and functional characterization of chalcone isomerase from *Carthamus tinctorius*

**DOI:** 10.1186/s13568-019-0854-x

**Published:** 2019-08-21

**Authors:** Xiuming Liu, Naveed Ahmad, Longyu Yang, Tianyu Fu, Jie Kong, Na Yao, Yuanyuan Dong, Nan Wang, Xiaowei Li, Fawei Wang, Xin Liu, Weican Liu, Haiyan Li

**Affiliations:** 1 Ministry of Education Engineering Research Center of Bioreactor and Pharmaceutical Development, Jilin Agricultural University, Changchun, 130118 China; 2 College of Life Sciences, Jilin Agricultural University, Changchun, 130118 China

**Keywords:** Chalcone isomerase (*CHI*), Flavonoids biosynthesis, Safflower, Expression analysis, HPLC

## Abstract

Flavonoid is one of the widespread groups of plant secondary metabolites that provide several health benefits. However, the explicit mechanism of flavonoid biosynthesis in plants largely remains unclear. Chalcone isomerase an important class of enzyme presents crucial role during flavonoid metabolism in many plants. Here, we isolated the full-length cDNA (1161 bp) of a novel *Chalcone Isomerase* from safflower encoding 217 amino acid polypeptide using oligos from 5′ and 3′ ends. The result of Sanger sequencing and phylogenetic analysis revealed that *CtCHI* is highly homologous to other plants, including typical polyadenylation signals AATAA and Poly A tail. The transient expression in tobacco mesophyll cells using Green Fluorescent Protein tagging determined the subcellular localization of *CtCHI* in cell membrane and nucleus. The *CtCHI* ectopic expression in different safflower varieties at different flowering stages showed that *CtCHI* were found in abundance at the bud stage of *Jihong No. 1*. Further correlation analysis between *CtCHI* expression and flavonoid accumulation at various flowering phases suggested that *CtCHI* might play a potential role during flavonoid biosynthesis in safflower. In addition, the overexpression of pBASTA-CtCHI in transgenic *Arabidopsis* infiltrated with floral dip transformation showed relatively higher expression level and increased flavonoid accumulation than wild type. Moreover, the in vitro enzymatic activity and HPLC analysis of transgenic *Arabidopsis* confirmed the de novo biosynthesis of Rutin. Taken together, our findings laid the foundation of identifying an important gene that might influence flavonoid metabolism in safflower.

**Electronic supplementary material:**

The online version of this article (10.1186/s13568-019-0854-x) contains supplementary material, which is available to authorized users.

## Introduction

**Fig. 1:**
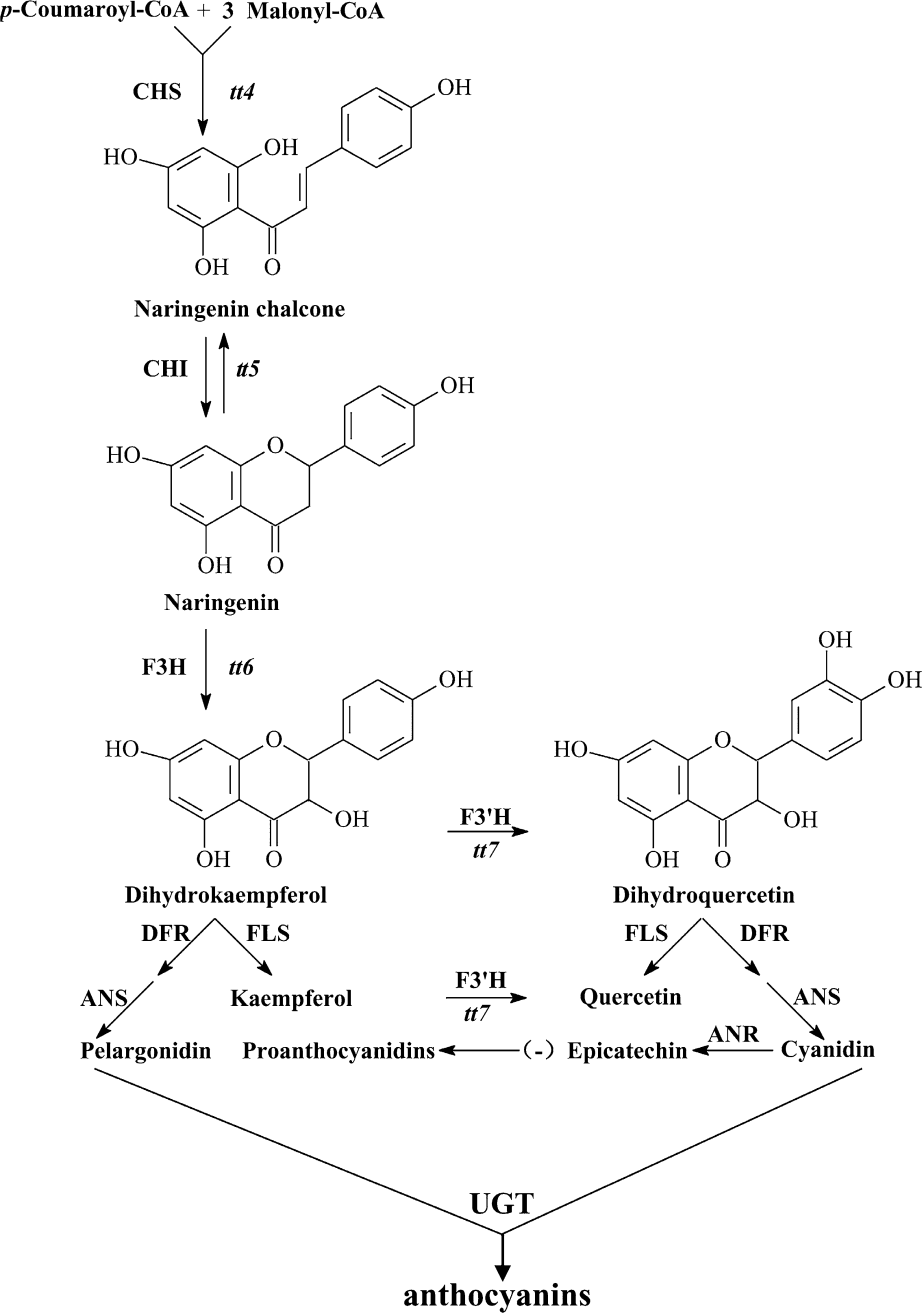
The core metabolic pathway of flavonoids (anthocyanins) occurred in *Arabidopsis thaliana* (Jiang et al. 2015)

Safflower (*Carthamus tinctorius* L. Compositae), a dicotyledonous plant, serves as an important traditional Chinese herb. It is not only used for medicinal purposes, but also as a food supplement in various kinds of nutritional oil. Safflower has been valued historically for its abundant Flavonoids content, fatty acids, various phenolic compounds, and lignin product (Dai et al. 2013). Flavonoids are one of the most important phenolic compounds (Guo et al. 2016; Yaginuma et al. 2003). Flavonoids are widely distributed class of plant producing secondary metabolites linked with a variety of metabolic functions in plants. Among secondary metabolites, the biosynthesis pathway used to produce flavonoid compounds is one of the most thoroughly elucidated metabolic pathways. In *Arabidopsis,* flavonoids are synthesized naturally by catalyzing *p*-coumaroyl-CoA and 3 malonyl-CoA molecules with the help of chalcone synthase (*CHS*) which is prearranged on *TT4* locus. The first product in this stepwise condensation manner is naringenin chalcone, which is then undergoing isomerization via chalcone isomerase (*CHI*) to form naringenin (Fig. 1). The second product (naringenin) is considered the basic intermediate of the flavonoid pathway. Following other metabolic routes during flavonoid metabolism naringenin is further converted to dihydrokaempferol with the help of flavanone 3-hydroxylase enzyme (*F3H*) encoded by *TT6* locus. Further stepwise hydroxylation of dihydrokaempferol can lead to the formation of dihydroquercetin by flavanone 3′-hydroxylase (*F3′H*). The aforementioned two important intermediates can be converted to other distinct groups of flavonoids such as anthocyanins, and flavonols and phenylalanines with the help of a complex group of multienzymes including anthocyanidin synthase (*ANS*), flavonol synthase (*FLS*), dihydroflavonol 4-reductase (*DFR*), UDP-glucosyltransferase (*UGT*) and anthocyanidin reductase (*ANR*) (Jiang et al. 2015; Meng et al. 2015).

Much effort has been given to elucidate flavonoid biosynthetic pathways from a genetic perspective. To date, most of the structural and several regulatory genes involved in the flavonoid biosynthesis pathway have been cloned in a variety of plants (Li 2014; Tu et al. 2016; Wang et al. 2016). The most important genes or their antisense sequences in the secondary metabolism of flavonoids have been transferred into other plant species through genetic engineering, leading to changes in the synthesis of flavonoid compounds by promoting or inhibiting the expression of these genes (Gutierrez et al. 2017). However, the biosynthesis pathway of flavonoids involves a multienzyme complex system including isomerases, reductases, hydroxylases, and several Fe^2+^/2-oxoglutarate-dependent dioxygenases, depending on the species involved. In addition, flavonoids play an important role in the plant response to adverse environments in vivo, for example, they can work as scavengers of free radicals such as reactive oxygen species (ROS) or being involved in the resistance of plants to drought (Jamalan et al. 2016; Kranner and Birtić 2005; Landry et al. 1995).

Recently, a number of CHI genes have been extensively characterized from different plant species (Dastmalchi and Dhaubhadel 2015; Jiang et al. 2015; Kang et al. 2014) and other functional genes were also identified during natural flavonoid biosynthetic pathway in *Carthamus tinctorius* including *CHS* (Guo et al. 2017), *UGTS* (Guo et al. 2016). However, there is no such report available on CHI from *Carthamus tinctorius* and is unreported up to date. Chalcone isomerase (CHI, EC:5.5.1.6), was found a key enzyme during flavonoid metabolic pathway in several other plants (Gensheimer and Mushegian 2004) which catalyze the isomerization of chalcones into their corresponding (−)-flavanones. CHI is commonly found in the form of monomers in most plant species with remarkably variable molecular weights. Genes of the CHI family generally encode proteins with 210–240 amino acids with a high frequency of conserved sequences. Moreover, the sequence homologies among different species are reported between 50 and 80%. Here, we presented the first report on the discovery and characterization of a new chalcone isomerase gene from safflower cultivar *Jihong No. 1* using an expressed sequence homology-based approach followed by subcellular localization in tobacco mesophyll cells through GFP tagging. In addition, the expression analysis in the transgenic *Arabidopsis* T3 homozygous plants was also carried out using real-time quantitative PCR analysis. For this purpose, the overexpressed lines of *Arabidopsis thaliana* harboring (PBASTA-CtCHI) construct were generated through floral dip transformation. Homozygous transgenic T3 plants were selected for further characterization, including expression analysis, in vitro enzymatic activity and HPLC analysis. Our findings implied the discovery of a novel chalcone isomerase gene which could induce the flavonoid biosynthetic pathway in *Carthamus tinctorius*.

## Materials and methods

### Strains, cells and experimental materials

Safflower seeds were provided by Fuyu seeds company, China. *Escherichia coli BL21 (DE3), Escherichia coli TransT1,* DH5α competent cells, and *A. tumefacien* strain EHA105 were purchased from Takara Biotechnology Company Beijing, China. Plant overexpression vector (pBASTA) and pCAMBIA1302-GFP-35S were purchased from TransGen.

Biotech, Beijing and preserved in our laboratory (Ministry of Education Engineering Research Center of Bioreactor and Pharmaceutical Development at Jilin Agricultural University, Jilin, China) until next use. Restriction enzymes, GºTaq DNA polymerase and DNA ligases were purchased from Takara Biotechnology Company Beijing, China.

### Plant materials

Four safflower cultivars, *Jihong* (early maturing line) (JHEM), *Jihongyou sister line* (JHS), *Jihong No. 1* (JH1), and *Jihong No. 2* (JH2) were grown at the experimental station of the Ministry of Education Engineering Research Center of Bioreactor and Pharmaceutical Development at Jilin Agricultural University, Jilin, China. Flower petals of each cultivar at the bud, initial, full, and fade stages were sampled and wrapped with tin foil, quickly placed into liquid nitrogen, and then stored at − 80 °C. Flower petals of each cultivar at the bud, initial, full, and fade stages were sampled and wrapped with tin foil, quickly placed into liquid nitrogen, and then stored at − 80 °C. Wild-type (WT) *A. thaliana* was grown for 5–6 weeks, and flowering plants were used for floral-dip transformation.

### Cloning and sequence analysis of *CtCHI*

The full-length cDNA sequence of *CtCHI* from *Carthamus tinctorius* was obtained by using 5′ and 3′ RACE PCR method following the initial denaturation at 94 °C for 6 min; 30 cycles of 94 °C for 45 s, 58 °C for 45 s, and 72 °C for 90 s; and a final extension step at 72 °C for 10 min. The appropriate pair of primer CtChir (AATAGGATCCCGGAAGTGCAATTACCAT) and CtChif (AATAGAATTCCTCGTGAAACTCCTGTTTTCT) containing restriction enzyme sites for *Eco*RI and *Bam*HI was designed using Premier 5.0 (Additional file 1: Table S1). The amplified PCR fragments were cloned into pEASY-T1 vector (Takara, Beijing) and then sequenced to investigate any base mutation. After confirmation of Sanger sequencing, the sequence assembly of *CtCHI* was performed, and the expected amino acid sequence was deduced using DNAMAN software (Lynnon Corp., Quebec, Canada) with default parameters. The maximum open reading frame was predicted by the Laser gene SeqMan II Module (http://www.DNAstar.com). The homologous sequences were aligned by BLASTX (with *E* value < 10^−10^) and compared against the National Center for Biotechnology Information (NCBI) database. The physicochemical characteristics of the *CtCHI* protein, including the amino acid sequence, relative molecular weight, and isoelectric point, were characterized using ProtParam software (http://web.expasy.org/). Homology analysis was carried out using DNAMAN software according to (Tamura et al. 2011).

### Determination of safflower yellow pigments at different flowering stages

Four different safflower varieties were selected at different flowering stages, including bud, initial, full, and fade stages. The flower petals were collected and dried in a thermostatic drier at 55 °C for 24 h. Each sample of the flower tissue was individually ground to a fine powder. The weight of 1 g powdered flower tissue was measured, followed by soaking in 25 ml of 50% methanol solution. The resultant solution was subjected to ultrasonic extraction using the following conditions: extraction temperature, 42 °C; extraction period, 30 min twice; centrifugation at 5000 rpm for 10 min. The supernatant was cautiously diluted in methanol and then infiltrated with a filter membrane (0.45-µm). The samples were then measured by comparing the absorbance values at 300 nm. Each experiment was performed in three independent biological replicates presented as means ± SE (n = 3). Asterisks indicate the statistical significance levels according to Student’s *t*-test: *P < 0.05.

### The interrelationship between *CtCHI* expression level and safflower yellow pigment accumulation during different flowering stages

In order to determine the correlation between *CtCHI* expression and accumulation of safflower yellow pigment, the expression level of *CtCHI* at four different flowering stages of *Honghua Jihong No. 1 (JH1)* cultivar was further investigated. The flower petals of the bud, initial, full, and fade stages were collected from *JH1* cultivar and then subjected to RNA extraction using RNA isoplus. The first strand cDNA templates were prepared by reverse transcription PCR. The expression of *CtCHI* gene was calculated by real time-PCR (qRT-PCR) analysis using primer pair RTCHI-F/R (Additional file 1: Table S1). qRT-PCR reactions were measured on (Applied Biosystems 7300/7500/7500 Fast Real-Time PCR System; Foster City, CA, USA). Each qPCR reaction was performed in a total volume of 20 μl, containing 10 μl SYBR Premix Ex Taq (Tli RNaseH Plus) (2×), 0.4 μl ROX Reference Dye II (50×), 0.4 μl of each of upstream and downstream primers (0.2 μM), 2 μl cDNA template (100 ng) and 6.8 μl ddH_2_O (Bio-Engineering Co., Ltd., Jilin, China. Item No. RR420A). The qRT-PCR thermal cycle was followed as initial denaturation (30 s at 95 °C), followed by 40 cycles of 30 s at 95 °C and 3 s at 65 °C. All reactions were carried out in 3 independent replicates, and the results were calculated according to 2^−ΔΔCt^ whereas 18 s ribosomal RNA gene (GenBank accession: AY703484.1) was used as a housekeeping gene.

### Subcellular localization of *CtCHI*

The subcellular localization of *CtCHI* was computationally predicted with the help of WoLF PSORT program (Horton et al. 2007). Subsequently, the *CtCHI* gene was amplified using the forward primer SLCHI-F (AAACTAGTATGGCTCCGCCGCCGTCCAC) and reverse primer SLCHI-R (AAAGATCTATTCATGAGATCGGCCAATC) containing restriction enzyme sites for *spe*I and *bgl*II. The amplified fragment of *CtCHI* gene was re-cloned into the linearized pCAMBIA1302 vector, which consists of a green fluorescent protein (GFP) under the control of the CaMV 35S promoter. After confirmation by sequencing, the recombinant product was transferred into *A. tumefaciens (EHA105)* via freeze and Thaw method. In the meanwhile, an empty vector of pCAMBIA1302 was also transformed into *A. tumefaciens.* Wild type tobacco plants were grown in a growth chamber (25 °C, 16 h light/8 h dark). Plants with at least two pairs of leaves were selected for infiltration. Prior to transient expression, the positive strains of *Agrobacterium* were selected and incubated in Luria–Bertani (LB) media containing 50 mg/l kanamycin and 50 mg/l rifampicin. The transformation was performed by injecting the harvested culture of *Agrobacterium* at an OD_600_ equal to 1.0 with the help of a syringe into the leaves of healthy wild tobacco plants. The GFP fluorescence of *CtCHI* protein and the pCAMBIA1302 empty vector were visualized using a laser confocal microscopy.

### Vector construction and *Agrobacterium*-mediated transformation in *Arabidopsis*

The full-length cDNA of *CtCHI* was ligated into a linearized plant over-expression vector (pBASTA). Before ligation, pEASYT1-CtCHI and empty vector of pBASTA was treated with double digestion system using *Bam*HI and *Eco*RI restriction enzymes. The digestion system was used as follow: Total volume of 20 μl reaction, containing 2 µg DNA, 2 μl of *Eco*RI and *Bam*HI, 2 μl Buffer K and ddH_2_O respectively. Ligation with the plant overexpression vector and *CtCHI* was performed using T4 ligase sticky end ligation system according to (Jailani et al. 2016). The pBASTA-CHI expression vector, along with the empty vector, was further transformed into EHA105 *E. coli* Trans (T1) competent cells. Positive colonies were detected by colony PCR. The *Agrobacterium*-mediated transformation of wild-type *Arabidopsis. thaliana* was carried out following the floral-dip infection method, according to (Jiang et al. 2015).

### Expression profiling of *CtCHI* gene in transgenic system

The transgenic *Arabidopsis* lines were grown in an artificial climate chamber up to a homozygous T3 generation. Transgenic plants were maintained in a controlled environment (25 °C, 16 h light/8 h dark) until harvesting. The leaves of 14 homozygous T3 lines were collected for the isolation of total RNA in order to synthesize the first strand cDNA via reverse transcription for qRT-PCR analysis, following the protocols described above.

### In vitro enzymatic activity of CtCHI

The double antibody sandwich ELISA was carried out to detect the relative amount of *CtCHI* analyte and its associated in vitro enzymatic activity obtained from the original samples of T3 homozygous *Arabidopsis* mutants. The samples with an unknown amount of *CtCHI* antigen were collected from 14 transgenic plants. The samples were immobilized for 30 min at 37 °C on a solid surface through a conjugation detection antibody (ELISA KIT, subpackage; RC9615 R&D Systems Inc America). The unbound antibodies were removed through washing using wash buffer. After final washing, the plate was subjected to conjugation with HRP Enzyme-linked secondary antibodies for 30 min at 37 °C in order to produce a visible signal. The plate was thoroughly rinsed with wash buffer to detach the unbound antibody-enzyme conjugates present in the samples and subsequently stained with a chemical 3,3′5,5′-tetramethylbenzidine (TMB) substrate to the wells resulted in blue color fluorescent indicating the amount of analyte present in the samples. The turning of blue color signals into yellow signals indicated the nonspecific reaction of HRP-labeled antibody. The development of the electrochemical signals (Blue/Yellow) obtained from 14 mutant *Arabidopsis* was measured as absorbance (OD) using a spectrophotometer at 300 nm to determine the quantity of *CtCHI* antigen in the samples.

### Determination of flavonoids accumulation in transgenic *Arabidopsis*

The leaves of transgenic *Arabidopsis* were collected and grinded, and then passed through a 40-mesh screen. Accurately weighted (0.5 g) of fine leaf powder was soaked in 50 ml methanol (65%) solution followed by sonication for 30 min with the following conditions: temperature 50 °C, ultrasonic wavelength 600 W with 40 kHz frequency. The resultant extract was carefully diluted in methanol solution and then purified through a filter membrane. After centrifugation at 12,000 rpm for 10 min, the supernatant was collected, filtered, and analyzed by HPLC analysis following the protocol of (Sun et al. 2016). The separation of Chromatic compounds was performed using different gradient solutions of the authentic standard of Rutin. Six different gradient solutions were prepared by dissolving 2, 4, 6, 8, 10, and 12 ml Rutin in 20 ml methanol. Chromatographic conditions includes: Column: Agilent zorbax SB-C 18 (4.6 mm × 150 mm, 5 μm); mobile phase: phase A is methanol–acetonitrile (V: V 1:10), phase B is 0.4% phosphoric acid solution. The gradient elution went from [94. 5% B → 86.8% B in 10 min, 20 min, 86.8% B → 81. 3% B in 20 min, 81. 3% B → 80. 75% B in 40 min, 80. 75% B → 76. 9% B in 40 min, 76. 9% B → 70. 3% B in 50 min]. Measured the detection wavelength at 340 nm; Column temperature: 25° C; at a Flow rate of 0.8 ml/min and Volume: 20 μl with three replicates.

## Results

### Cloning, sequence analysis and characterization of the *CtCHI* gene

**Fig. 2:**
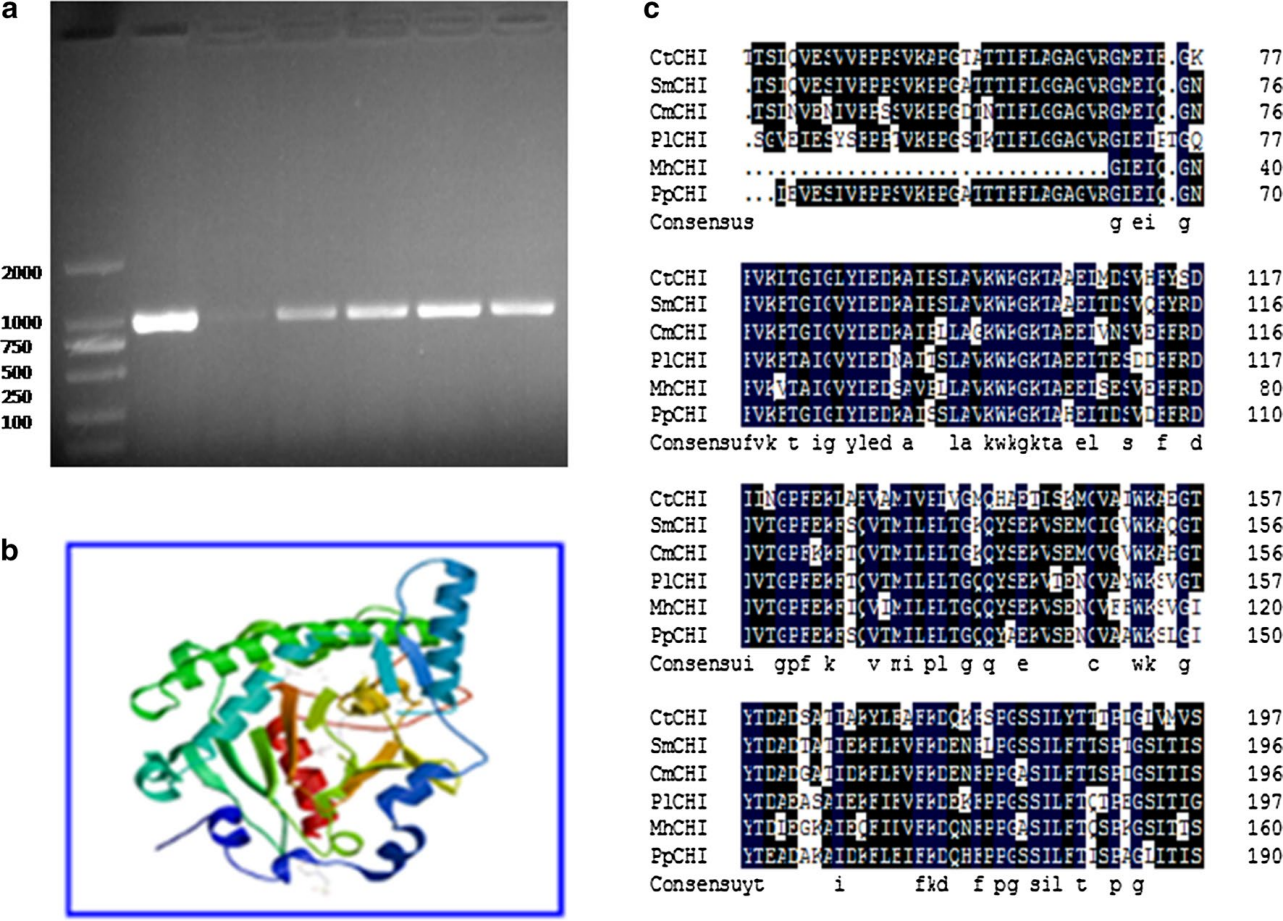
Cloning and sequence analysis of the *CtCHI* gene. **a** cDNA of *CtCHI*. **b** Tertiary 3D structure of the *CtCHI* protein. **c** Alignment of the deduced amino acid sequence of *CtCHI* gene with other plant species. *CtCHI*: *Carthamus tinctorius* L.; SmCHI: *Saussurea medusa*; pl CHI: *Paeonia lactiflora*; Mh CHI: Malus hybrid; Pp CHI: *Pyrus pyrifolia*

We previously analyzed the high throughput transcriptomic analysis obtained from RNA sequencing of safflower petals in order to discover the Safflower yellow pigment biosynthetic related genes (Liu et al. 2015). In the current study, the full-length cDNA (1161 bp) of the *CtCHI* gene (GenBank accession: KP300882) was cloned using 5′ and 3′ rapid amplification (Fig. 2a). Sequence analysis showed that *CtCHI* contained an open reading frame of 654 bp encoding a polypeptide with 217 amino acids. It was found that the length of 5′ untranslated region (5′ UTR) consist of 250 bp while the 3′ untranslated region (3′ UTR) was found 257 bp including a typical Poly (A) tail. Three-dimensional protein structure prediction confirmed that the protein encoded by *CtCHI* gene was a monomer with 23.14 Ku of theoretical molecular weight and had an isoelectric point (pI) of 5.67 which is somewhat homologous (53.52%) to the template proteins in the SWISS-MODEL database (https://swissmodel.expasy.org/repository) (Fig. 2b). BLASTN query from the NCBI database confirmed the grouping of *CtCHI* to chalcone superfamily. Further phylogenetic analysis revealed the comparative homology of the *CtCHI* gene with other *CHIs* from other five dissimilar species including dahlia (65.97%), chrysanthemum (66.17%), apple (50.99%), *Aristolochia debilis* (69.17) and peony (69.55%). Based on the phylogenetic divergence exhibited by *CtCHI* gene in these uncommon species confirms the hypothetic role of a new *Chalcone isomerase* in *Carthamus tinctorius* (Fig. 2c).

### Measurement of safflower yellow pigment at various flowering periods

**Fig. 3:**
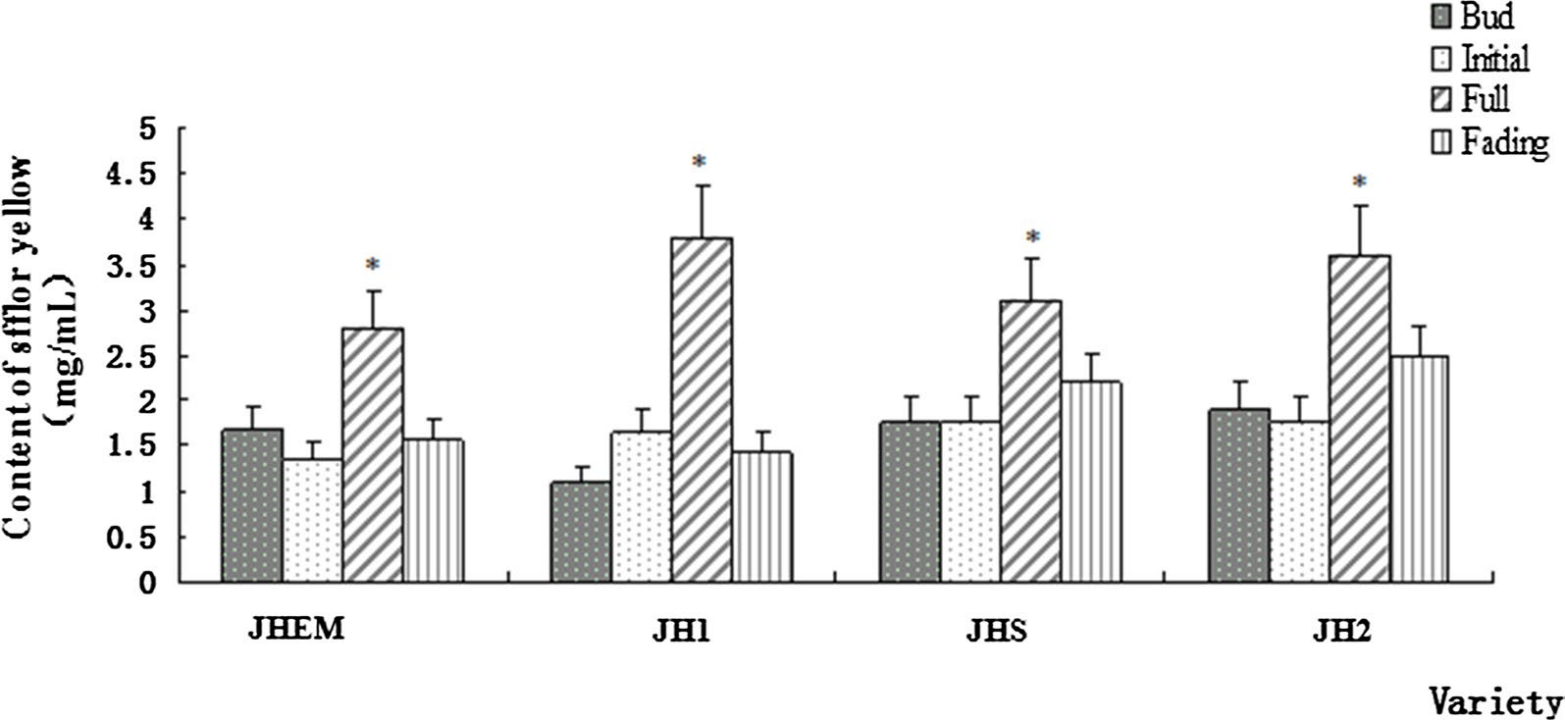
The content of yellow pigment of safflower accumulated during four different flowering periods in four different varieties. Safflower varieties were labeled as JHEM, JH1, JHS, and JH2. Different flowering periods were indicated as a bud, initial, full, and fading stage. Data are means ± SE (n = 3). Asterisks indicate the statistical significance levels according to Student’s *t*-test: *P < 0.05

The yellow pigment of safflower was extracted from four different varieties of *Carthamus tinctorius* including (*JHEM, JHI, JHS, and JH2*). The contents of Safflower yellow pigment at four different flowering stages consisting of bud, initial, full, and fade stage were determined using spectrophotometric measurement, respectively (Fig. 3). A unique pattern of the ultra-violet wavelength at 300 nm was strikingly absorbed for the yellow pigment accumulation during all four varieties. However, the amount of yellow pigment during the full flowering stage was found significantly higher than the other three stages in each variety. Out of the four varieties, JH1 had the highest amount of yellow pigments synthesis occurred during the full stage, reaching 3.791 mg/ml. Therefore, JH1 was used as the experimental object in our study.

### Correlation between *CtCHI* transcript abundance and SYA accumulation in safflower

**Fig. 4:**
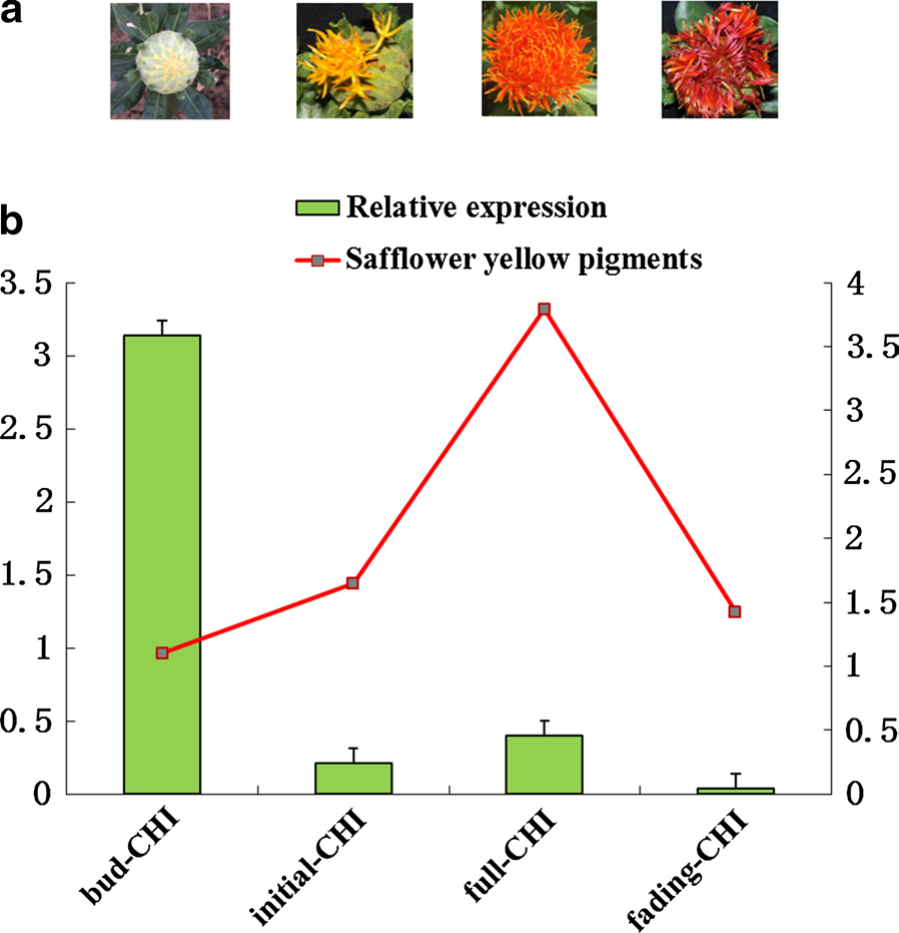
The correlation between expression of *CtCHI* gene and biosynthesis of flavonoids at various flowering stages of safflower. **a** Different flowering stages of safflower; **b** RT-qPCR transcript levels of *CtCHI* gene which are indicated in bars while the synthesis of safflower yellow pigment at various developmental stages are presented as red letter boxes. The 18s ribosomal RNA gene was used as an internal control. Error bars indicate the SE (n = 3)

After the selection of *JH1 safflower* cultivar displaying the maximum accumulation of safflower yellow pigment, we further investigated the correlation between the transcript abundance of *CtCH1* gene and safflower yellow pigment accumulation that took place at different flowering stages using qRT-PCR analysis. It was observed that the expression level of *CtCHI* gene at bud stage was found greater than the other three stages. However, the yellow pigment accumulation was consistently remained higher at the full stage with the notably decreased expression level of *CtCHI* than in bud, initial, and fade stages (Fig. 4). Moreover, the accumulation pattern of safflower yellow pigment at *CHI*-Initial and *CHI*-fading stages were appeared to synthesize a relatively similar amount of yellow pigment. Nevertheless, the expression pattern of *CtCHI* showed a reverse order, suggesting the metabolon mechanism of *CtCHI* during both of these stages occurred from the similar upstream gene, or it may be the result of a synergistic consequence on safflower yellow pigment metabolism at this specific junction. Furthermore, the accumulation of a moderate level of yellow pigment at CHI-bud stage with the relatively increased expression level of *CtCHI* at this stage indicated the importance of *CtCHI* gene during flavonoid synthesis in *Carthamus tinctorius*.

### Subcellular localization of *CtCHI* using GFP tagging

**Fig. 5:**
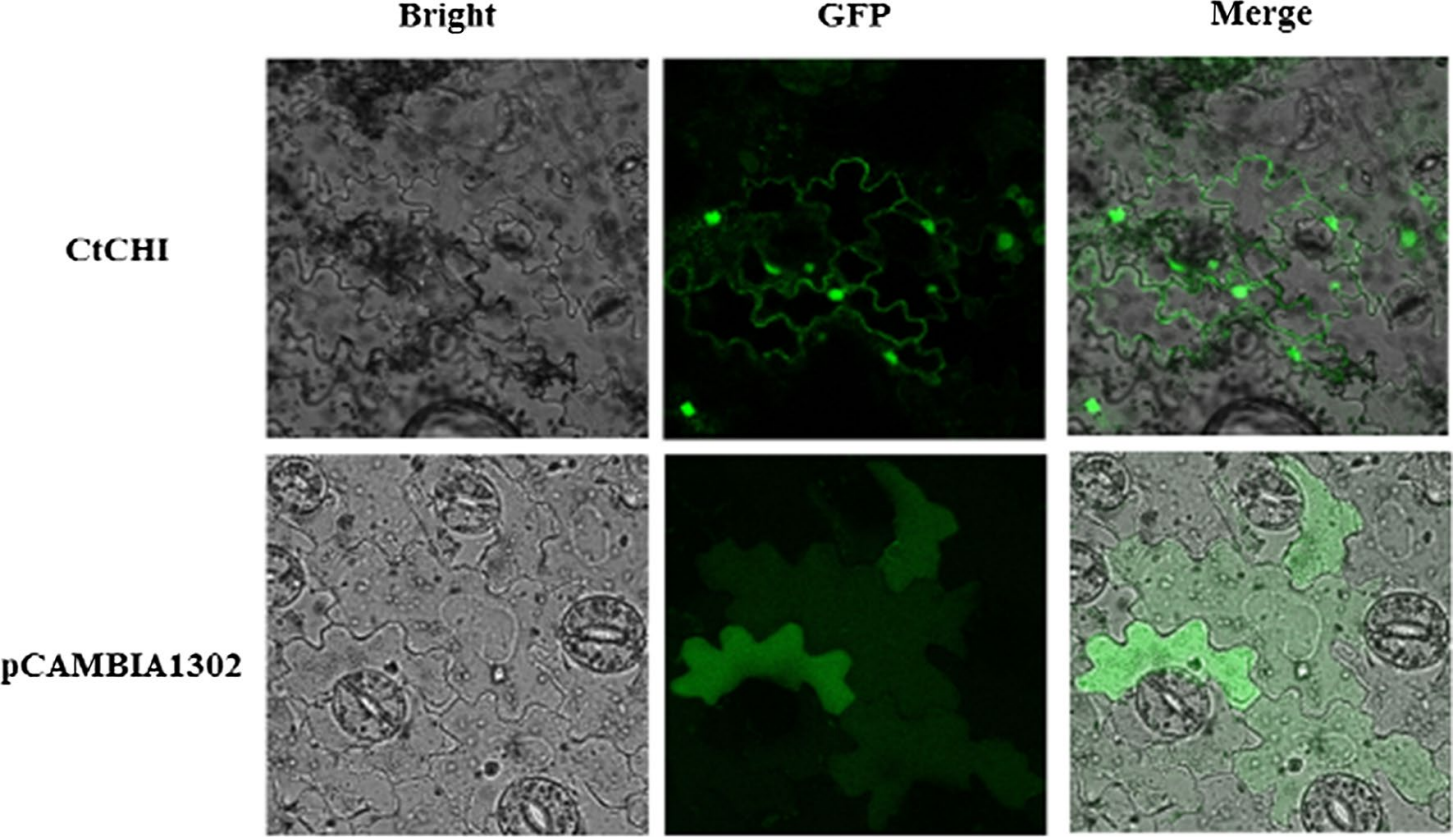
Subcellular localization of CtCHI-GFP in wild type tobacco mesophyll cells. GFP signals of *CtCHI*-pBASTA1302-GFP fusion construct localizes to both cytosol and nucleus. The pBASTA1302-GFP signals were detected dispersed in tobacco mesophyll cells. The GFP fluorescence exhibited signals were analyzed with a confocal laser scanning microscope

The theoretical prediction of *CtCHI* protein revealed a 60.4% probability to be located in the nucleus and 17.5% of probability localization in the cytoplasm. Then, we investigated the precise subcellular localization of *CtCHI* gene through Green fluorescent protein (GFP) tagging using a fusion construct of pCAMBIA1302 vector and *CtCHI* under the control of CaMV 35S promoter. In this report, the pCAMBIA1302 empty vector was used as a control. Wild type tobaccos were infected with pCAMBIA1302-*CtCHI and* pCAMBIA1302 alone via *Agrobacterium* (EHA105) mediation. The GFP fluorescence of *CtCHI* gene and the empty vector was analyzed under a confocal laser scanning microscope. As described in Fig. 5, the tobacco plants harboring the *CtCHI*-pBASTA1302-GFP vector exhibited GFP signals predominately in nucleus and cytoplasm. On the contrary, tobacco carrying pBASTA1302-GFP alone was detected solely inside mesophyll cells. These findings indicated that *CtCHI* was originally localized in the nucleus and therefore could possibly regulate a physiological pathway in *Carthamus tinctorius*.

### Identification of *CtCHI* overexpressed T3 *Arabidopsis*

Transgenic seeds were grown up to T3 generation, and then selection and screening of the homozygous T3 transgenic plants were performed by spraying 1% Basta (Additional file 1: Fig. S3). The use of antibiotic selection could eliminate several drawbacks when generating and identifying transgenic plants simply by removing fungal contamination. It can also help to screen out those lines which were internally contaminated by the *Agrobacterium* strain itself. Further identification of the mutant plants was carried out using the BAR gene as a selectable marker to detect the presence of transgene pBASTA-CtCHI (Additional file 1: Fig. S1). Additionally, we examined the presence of terminator gene (NOS) in the selected transgenic lines of *Arabidopsis* (Additional file 1: Fig. S2). For this purpose, the genomic DNA of transgenic plants was extracted with the help of NuClean plant Genomic DNA Kit (Beijing, China). PCR amplification of *CtCHI*, BAR gene, and NOS terminator gene was performed respectively using GºTaq DNA polymerase (Promega Corp, Madison, WI) with the help of the respective pair of primers (Additional file 1: Table S1).

### Quantitative real-time expression and in vitro activity of *CtCHI* in transgenic *Arabidopsis*

**Fig. 6:**
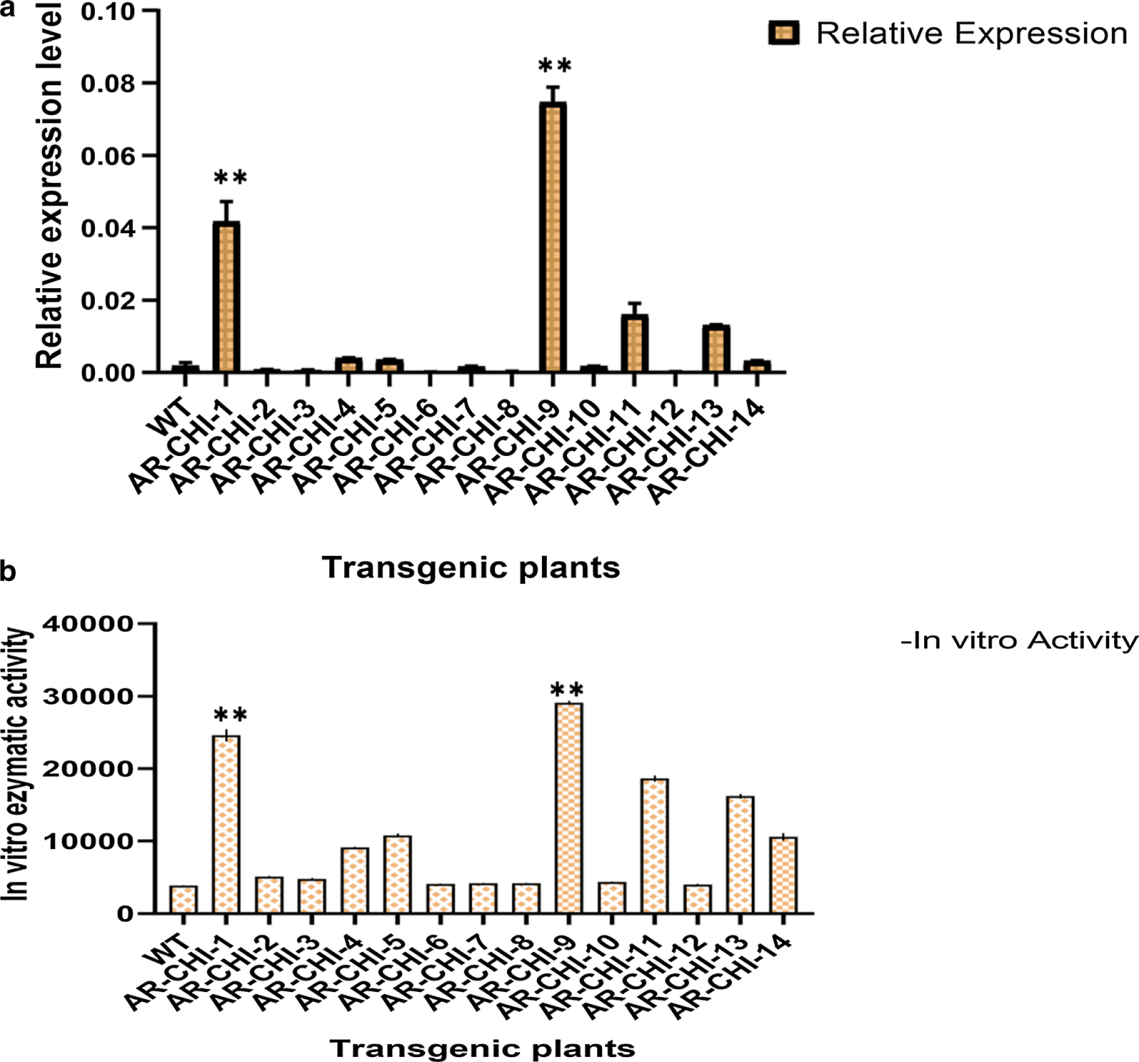
Quantitative real time Expression levels of *CtCHI* gene and CHI activity in transgenic *Arabidopsis* lines. **a** Relative expression levels of *CtCHI* gene presented in four different transgenic lines in comparison with the wild type *Arabidopsis*. The data were normalized with 18s ribosomal RNA. **b** In vitro enzymatic activity of *CHI* using ELISA kit, RC9615, R&D Systems Inc., America). The color of the 3,3′,5,5′-tetramethylbenzidine was measured as absorbance (OD) using a spectrophotometer at 450 nm to calculate the concentration of CHI activity according to a standard regression curve. Data were presented as the mean ± SD. **P < 0.01 vs. the wild type group

The leaves of 14 homozygous *Arabidopsis* T3 lines and WT plants of *Arabidopsis* were collected and subject to RNA extraction for synthesizing the template cDNA through reverse transcription PCR. The expression level of the *CtCHI* gene in the transgenic *Arabidopsis* lines was normalized using 18S ribosomal RNA as an internal reference gene in the qRT-PCR system. According to Fig. 6, the expression analysis of 14 transgenic lines showed a correspondently higher level of *CtCHI* mRNA transcript abundance accumulated in the *AR*-*CHI*-*9 followed by AR*-*CHI*-*1* mutant line in comparison to other transgenic and wild-type plant. According to the core metabolic biosynthetic pathway in safflower, the expression of *CtCHI* should be negatively correlated with metabolite biosynthesis, however, the opposed pattern of *CtCHI* mRNA transcripts in *AR*-*CHI*-*9 and AR*-*CHI*-*1* transgenic systems implied the potential role of *CtCHI* during metabolite biosynthesis.

Furthermore, to investigate the interrelationship of *CtCHI* expression level and *CtCHI* in vitro enzymatic activity, we measured the relative mass values of the natural analyte present in the selected homozygous T3 lines using double antibody sandwich ELISA method (see “Materials and methods” for details). The potential of R&D Systems to determine the supreme standard curve range for individual sample by asserting results of peak sensitivity and reproducibility. Our findings indicated the increased *CtCHI* activities in two transgenic *Arabidopsis* lines (*AR*-*CHI*-*9* and *AR*-*CHI*-*1*), which exhibited the highest electrochemical signals resulted in blue color visualization at absorbance (OD) equals to 450 nm. It was found noteworthy that the expression level of *CtCHI* transcript abundance in the transgenic systems of *AR*-*CHI*-*9* and *AR*-*CHI*-*1* were found consistent and with a parallel manner to their in vitro enzymatic activities, as shown in Fig. 6. However, the lower CHI expression in other transgenic plants was not likely associated with in vitro activity of *CtCHI*, and likely have to do with procyanidin or other types of pigments present early maturation phases. Based on these findings, we speculate the partial function of *CtCHI* providing adequate insights during flavonoid biosynthesis.

### HPLC analysis in transgenic *Arabidopsis*

**Fig. 7:**
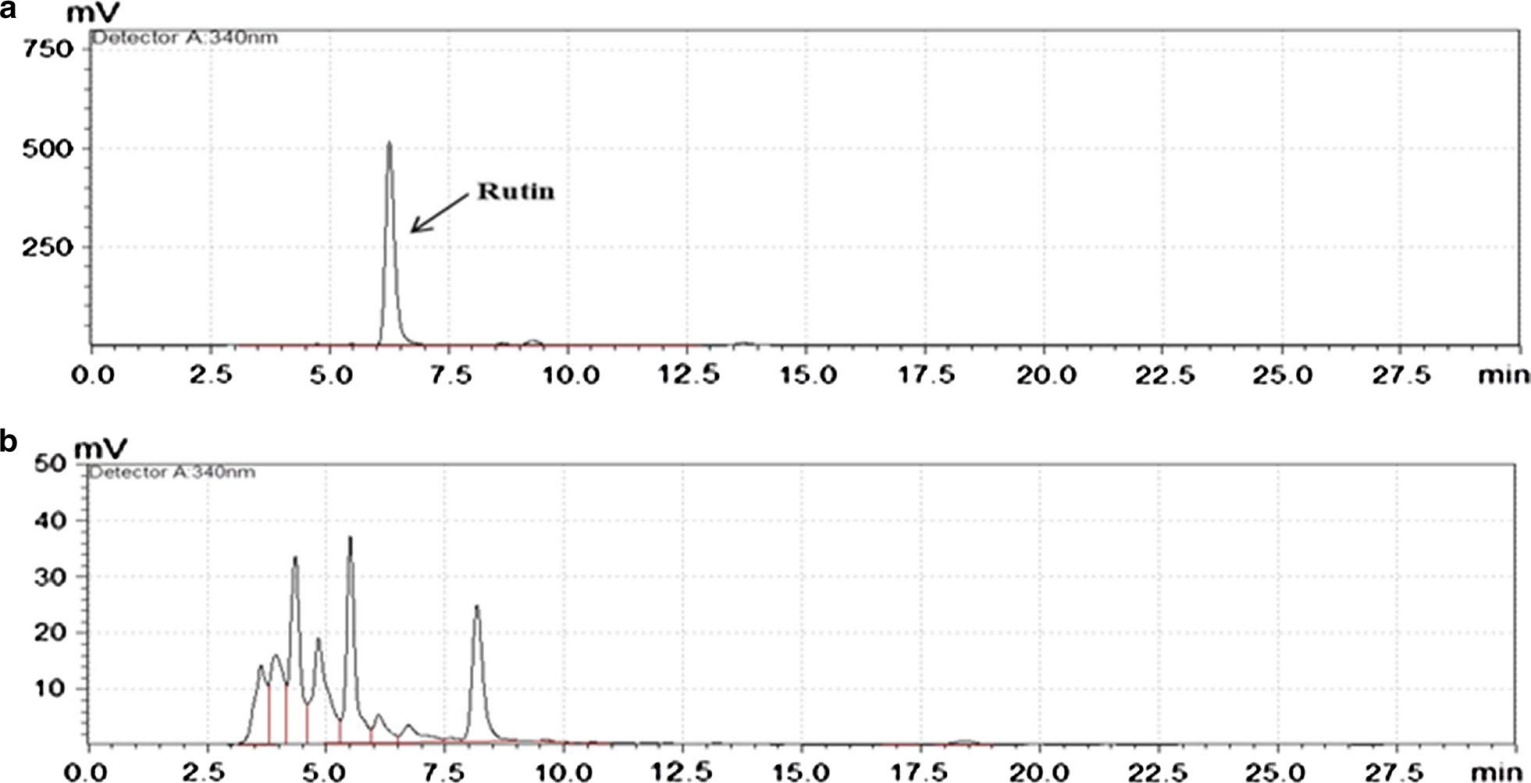
High performance liquid chromatography profile of Rutin in transgenic *Arabidopsis* lines purified through column Agilent zorbax SB-C 18 (4.6 mm × 150 mm, 5 μm); Mobile phase A as methanol–acetonitrile (V:V 1:10) and phase B as 0.4% phosphoric acid. **a** The peak labeled as Rutin was used as a reference in our study. **b** Chromatogram of high performance liquid chromatography of Rutin in transgenic *Arabidopsis*

Wild-type *Arabidopsis* plants normally display a mixture of quercetin and kaempferol glycosides. The standard peaks of these compounds were identified (data not shown) and compared with solvent extract peaks obtained from transgenic *Arabidopsis* T3 plants expressing the putative CHI gene. According to the standard curve of linear regression, the accumulation of increased quercetin-glycoside (rutin) reaching up to (187.37 μg/mg) in transgenic *AR*-*CHI*-*9* line followed by *AR*-*CHI*-*1* (173.67 μg/mg) is noteworthy as shown in the HPLC chromatogram (Fig. 7). The increased tendency of Rutin accumulation in *AR*-*CHI*-*9* and *AR*-*CHI*-*1* showed a similar pattern in comparison to *CtCHI* relative expression and in vitro CHI activity in these lines. Altogether, these results highlight the identification of a new *CtCHI* from *Carthamus tinctorius* promoting flavonoid biosynthesis.

## Discussions

Chalcone isomerase is one of several important enzymes in the biosynthesis of flavonoids, catalyzing the stereospecific isomerization of chalcones into their corresponding (−)-flavanones catalysis (Cheng et al. 2011). Several studies have demonstrated the roles of the CHI gene in the regulation of plant metabolism and changes in Flavonoid components in flower petals (Nishihara et al. 2005). However, the explicit expression pattern of CHI gene in flavonoid metabolic pathway in safflower remains unclear. In this study, the full-length cDNA of *CtCHI* gene was cloned from safflower petals using reverse transcription polymerase chain reaction. Blast result and phylogenetic analysis revealed that the CHI gene is highly homologous to *CHI* gene of the other plant species, indicating that this gene is highly conserved in plants which were consistent with the results of (Hong et al. 2012). The biosynthetic class of enzymes resides majorly in the nucleus where they play an essential role as DNA protectants against Ultra-Violet radiation as well as they are capable of regulating other related genes in one way or another which are necessary for other important physiological processes (Polster et al. 2006). Subcellular localization analysis localized the *CtCHI* gene on the cell membrane and in the nucleus, disagreeing with the previous results of (Dastmalchi and Dhaubhadel 2015), who reported the CHI gene on the nucleus and in the cytoplasm of soybean. Differences of the chromosomal locations of this gene might vary among plant species (Lv et al. 2017).

The in vitro enzymatic activity of *CtCHI* in *AR*-*CHI*-*1* and *AR*-*CHI*-*9* transgenic *Arabidopsis* lines exhibited the highest activity in catalyzing the synthesis of Rutin than all mutant plants and wild type plants. Additionally, the expression pattern of *CtCHI* in these plants was also consistent with it’s in vitro activity, which implies the positive role of *CtCHI* in flavonoids metabolism in safflower. Chalcone isomerase gene has been proven necessary for the biosynthesis of flavonoids and other essential pigments (Morita et al. 2014). We, therefore, analyze the expression levels of the *CtCHI* gene which indicates significant differences at various stages of flower development in safflower, peaking at the bud stage when the amount of pigments is the lowest and declining at the full stage when the content of pigments was at peaks. The result provides considerable evidence for the partial function of CHI gene during the regulation of floral pigmentation but the expression levels of *CtCHI* didn’t significantly correlate with pigments content, speculating that at the bud stage the flower color undergoes from faint yellow to yellow, while the expression of the CHI was relatively low in the more stable full stage.

The CHI gene is the main structural gene encoding the most important rate-limiting enzyme in the biosynthetic pathway of flavonoids. We also identified other putative CHIs in safflower (Additional file 1: Fig. S4) however, the expression level of these putative CHIs showed relatively lower expression level in comparison to *CtCHI* at the bud stage of JH1 cultivar. Previous studies have shown that the expression of the CHI gene in plants greatly influence flavonoid biosynthesis (Jiang et al. 2015; Nishihara et al. 2005; Wang et al. 2015). In many plant species, mutations with a deletion of the CHI gene may limit some steps in flavonoid biosynthesis, leading to a significant decrease in the content of flavonoids and anthocyanins produced (Kim et al. 2004; Van Tunen et al. 1988). In the present study, the certain increase of the amounts of flavonoids was detected in two transgenic *Arabidopsis* lines, consistent with previous studies (Muir et al. 2001; Park et al. 2011; Zhang et al. 2009). The result of this study provides supportive evidence for an active relationship between CHI gene expression and flavonoid synthesis. By cloning a CHI gene from *Saussurea medusa* and thereby over-expressing it in transgenic tobacco, (Li et al. 2006) reported that transgenic plants produced up to fivefold more total flavonoids over WT plants. Overexpression of the CHI gene in transgenic plants is an effective method for enhancing the content of flavonoids. In our findings, however, the number of flavonoids produced in transgenic *Arabidopsis* lines was maximally increased by twofold over WT plants. This has occurred because CHI is a rate-limiting enzyme (Muir et al. 2001) at the upstream of the flavonoid biosynthetic pathway, during which many important enzymes are involved in the regulation of the complex process of flavonoid biosynthesis. Over-expression of *CHI* may stimulate the expression of other key enzymes to facilitate flavonoid metabolism. In this study, *AR*-*CHI*-*9* had the highest yield of flavonoids than other transgenic *Arabidopsis* mutants. This might occur as a result of differential gene expression pattern influenced by different environmental factors or other internal reasons; this could be further verified by phenotypic experiments in future researches.

## Additional file

**Additional file 1: Table S1.** List of primers. **Fig. S1.** Plant over expression vector. **Fig. S2.** Detection of transgenic plants. **Fig. S3.** Strongest transgenic Arabidopsis lines obtained in our study. **Fig. S4.** Other putative CHIs in safflower. **Fig. S5.** Graphical representation of pCAMBIA1302-GFP-35S vector.

## Data Availability

All the data which is generated and analyzed during our study are included in the article and additional files.

## Abbreviations

GFP: green fluorescent protein
HPLC: high performance liquid chromatography
PI: isoelectric points
kDa: kilo dalton
CDS: coding sequences
qRT-PCR: quantitative real-time PCR
MW: molecular weight
JH1: *Jihong No. 1* (safflower cultivar)
WT: wild-type
CaMV: cauliflower mosaic virus
LB: Luria–Bertani
ELISA: enzyme-linked immunosorbent assay
