# Supplementary material for: Molecular cloning and functional characterization of chalcone isomerase from Carthamus tinctorius
Source: AMB Express. 2019 Aug 21;9:132. doi: 10.1186/s13568-019-0854-x (PMC6704227; doi:10.1186/s13568-019-0854-x)
Supplement: Supplementary file 1 — Additional file 1: Table S1. List of primers. Fig. S1. Plant over expression vector. Fig. S2. Detection of transgenic plants. Fig. S3. Strongest transgenic Arabidopsis lines obtained in our study. Fig. S4. Other putative CHIs in safflower. Fig. S5. Graphical representation of pCAMBIA1302-GFP-35S vector. [file 13568_2019_854_MOESM1_ESM.docx]

**Additional File**

**Tab. S1**

**List of primers**

The list of all primers used during our study.

| Primer | Primer sequences (5′-3′) | Use |
| --- | --- | --- |
| CtCHI-RF | F:AATAGAATTCCTCGTGAAACTCCTGTTTTCT  R: AATAGGATCCCGGAAGTGCAATTACCAT | Cloning of the 5′ and 3′ rapid amplification of cDNA ends of the *CHI* |
| RTCHI-F/R | F:AAGATACTTGGCAATGGTTGCG  R:CGGTATGCAACATGCCGAA | Fluorescence quantitative PCR primers |
| SLCHI | F (AAACTAGTATGGCTCCGCCGCCGTCCAC  R (AAAGATCTATTCATGAGATCGGCCAATC | Subcellular localization |
| 18S | F:GAGAAACGGCTACCACATCCAA  R:TCGTTTGAGCCCGGTATTGTTA | Reference gene in qRT-PCR analysis |
| BAR-N | F: TCAAATCTCGGTGACGGGC  R: GTCTGCACCATCGTCAACCACTA | Detection of transgenic lines |
| TNOS-X | F:GAATCCTGTTGCCGGTCTTG  R: TTATCCTAGTTTGCGCGCTA | Detection of transgenic lines |

**Fig. S1**

**Plant over expression vector**

The schematic diagram of plant overexpression vector (pBASTA) used during floral dip infilteration.

**Fig. S2**

**Detection of transgenic plants**

(A) PCR amplification of the BAR gene in transgenic lines (B) Detection of NOS terminator.

(C) The efficient amplification of CtCHI was obtained using gene specific primer.


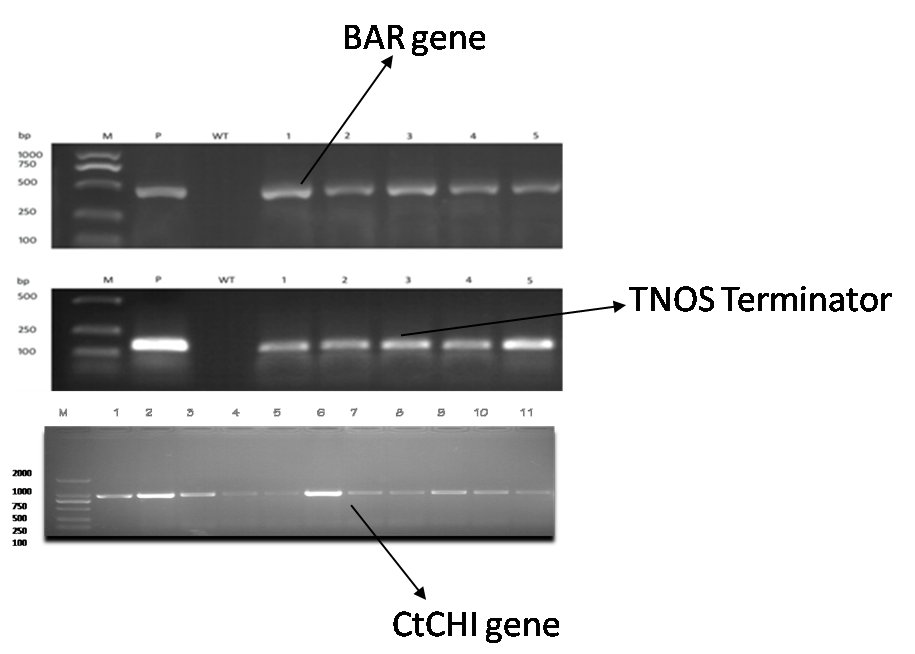


**Fig. S3**

**Strongest transgenic Arabidopsis lines obtained in our study**

The CtCHI overexpressed transgenic Arabidopsis homozygous T3 plants.


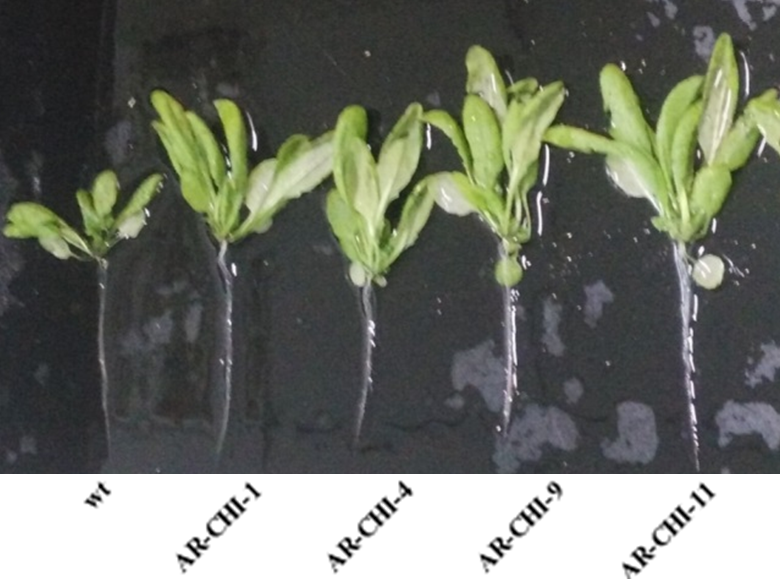


**Fig. S4**

**Other putative CHIs in safflower**

The complete list of CHIs in safflower other than CtCHI were also identified. The physicochemical properties of each gene including gene id, locus and ORF are given below.

>CCG012870.2 [mRNA] locus=scaffold_296:254066:254760:+ 663bp

ATGGCAGCTCTGACGCCGTCCTCCACCAGCATCCAGGTCGAATCCATCGTCTTTCCGCCCTCCGTCAAGCCTCCCGGCGCCACCACCACTCTATTCCTAGGAGGCGCAGTAATAGCGGCGTCGAACCCACGAACACACACGCACGCACGTCTCCCCGGCAGTATCAGCATCTACGGTCAGCCCACTAATCCGTCGCCGTCCTGGTCCACACGCTGGACCCAGAGTCCAACCACAGCCTTTGACTGGGCTGCAACTGGGCTAAGGGCAGCCCAGTCTATGACTGGACAAGCCCAAGGACTGCATCATGCCCTCAAATTGACTCCTCTTTGGATTCGAACTGGAATCCCATCCACACTCCACGGATGTTACCTGTTACCGCCCTTAGGAAGACCAACTGAACTAGACATGATTTGTGGTCTGGTGATATATGTATTGGAGTTTGAATCGTGTTTCTATGGTGCGCAAGGTGTGAGAGGTCTGGAGATCGAAGGTAGGTTCGTGAAGTTTACCGGAATCGGTGTGTATTTGGAGGATAAAGCTATTCCGTCACTCGCCGGCAAGTGGAAGGGGAAAACCGCTGCTGAGCTGACGGATTCCGTCCAGTTCTTCAGGGACATCGTTACCGGTAATACTCAACAGCTTATTATACTTCTAAGTTATTGA

>CCG022006.1 [mRNA] locus=scaffold_527:431453:433720:- 654bp

ATGGCTCCGCCGCCGTCCACCACCACCAGTCTTCAGGTCGAATCCGTCGTCTTTCCGCCATCCGTCAAGGCCCCGGGCACAGCCACCACTTTGTTCCTTGCAGGCGCAGGTGTGAGAGGTATGGAAATCGAAGGCAAGTTCGTCAAGTTAACGGGGATTGGCCTGTATTTAGAGGATAAAGCCATTCCGTCACTCGCCGTTAAGTGGAAGGGCAAAACCGCGGCTGAGTTGATGGACTCCGTCCACTTCTACAGCGACATCATTAATGGCCCCTTTGAAAAACTTGCCGAGGTGGCAATGATAGTACCATTAGTCGGTATGCAACATGCCGAAACGTTGTCTAAAATGTGCGTTGCGATTTGGAAAGCGGAGGGGACCTATACCGATGCAGATTCCGCAACCATTGCCAAGTATCTTGAGGCTTTTAAGGACCAAAAGTTTTCACCTGGCTCCTCTATTCTCTACACAACAACGCCCGATGGATTAGTAATGGTTAGCTTTGTGAAAGATGGTATCATACCTGAAACTCCGATTGTCGTGTTAGAGAACAAAAAACTGGGACACGCATTATTCGAGTCCGTGATCGGGAAGAATGGTGTTTCTCCTGAAGCGAAACAAAGTTTGGCATCAAGATTGGCCGATCTCATGAATTAG

>CCG031239.1 [mRNA] locus=scaffold_94:892980:896497:- 717bp

ATGGACTCGGCAGCACCGCGGCATGGCCTCAGTACCGAACCGGTACCCTCGGCAACACCGCCGAGGGTGACCGGCACGGTAGCCGTGGGATCAGAAATGGTGATGGTGGATGATATCCCTTTTCCTTCAGAGATCACAACTACCAAGCCTTTATCTTTGCTTGGACATGGAATTACCGACATCGAGATACATTTTTTGCAAATTAAGTTCACTGCCATCGGAGTTTATATCGACCCCGAGATCGTGGCTCATTTGCAAAAGTGGAAGGGTAAATCCGGAACCGAGTTAGCCGAAGATGATGAGTTCTTTGATTCTGTTATTTCGGCACCGGTCGATAAGTACTTGAGAATTGTGACAATCAAGGAGATCAAGGGATCGCAGTACGGGGTACAACTTGAGAGCTCGGTTAGGGACCGATTGGCGGCGGATGACAAGTATGAAGAGGAGGAGGAGGCCGCCCTCGAACAAATTGTAGAGTTCTTTCAATCGAAATATTTCAAAAAGGATTCGGTTATTACGTTTAGCTTTCCCGCAACATCAAACGTTGCAGAGATTGGGTTCTCAAGTGAAGGAAAAGAAGAGCCAAAGACACTGAAAGTGGAAAACGGGAATGTGGTGGAAATGCTGAAGAAATGGTACCTCGGTGGCACGAGTGCTTACTCCCCATCGACCATTTCATCATTGGCCAACACGTTGTCTTTGAAGTTGTCTAAATAA

**Additional figure**

**Fig. S5**

**Graphical representation of pCAMBIA1302-GFP-35S vector**

The schematic diagram of pCAMBIA 1302 vector used for the identification of CtCHI subcellular localization in tobacco leaves.
